# Turning problems into progress for primary care research trainees: a mixed-methods analysis of an online cross-sectional survey

**DOI:** 10.3389/fmed.2026.1786438

**Published:** 2026-05-15

**Authors:** K. Taylor Bosworth, Anna Walsh, Geetika Gupta, Chloe L. Warpinski, Meghan Gilfoyle, MaCee Boyle, Pamela Padilla, Kimberley Norman, Bryce Ringwald

**Affiliations:** 1 University of Missouri School of Medicine, Columbia, MO, United States; 2 Department of Psychiatry, University of Missouri School of Medicine, Columbia, MO, United States; 3 Faculty of Medicine, Centre for Rural Health Studies, Memorial University of Newfoundland, St. John’s, NL, Canada; 4 Faculty of Medicine, Dentistry and Health Sciences, University of Melbourne, Melbourne, VIC, Australia; 5 College of Medicine, University of Florida, Gainesville, FL, United States; 6 Department of Anthropology, College of Liberal Arts and Sciences, University of Florida, Gainesville, FL, United States; 7 Women’s College Hospital Institute for Health System Solutions and Virtual Care, Toronto, ON, Canada; 8 San Diego State University Research Foundation, San Diego, CA, United States; 9 School of Primary and Allied Healthcare, Monash University, Melbourne, VIC, Australia; 10 Bon Secours Mercy Health St. Rita’s Medical Center, Lima, OH, United States

**Keywords:** advocacy, barriers and facilitative factors, primary care, primary care research, research training and mentoring, trainee, trainee-led, workforce and research

## Abstract

**Background:**

As the field of primary care research continues to grow, it is increasingly important to address the concerns of our trainees. Trainees are central to workforce development and represent the future of the field. Identifying the specific needs and barriers they face in pursuing primary care research is essential to advancing the discipline.

**Methods:**

In this mixed-methods approach, we analyzed responses to an 33-item cross-sectional survey via REDCap. We performed quantitative analysis using RStudio for Windows (version 2025.05.1) and manually coded overarching themes in Microsoft Excel using an inductive thematic analysis approach.

**Findings:**

Sixty-nine survey responses were included in the quantitative analysis. A majority of responses were from allopathic medical (MD) students, representing 28.08% of respondents (*n* = 18), followed by medical residents (*n* = 17; 24.64%). We received responses from four countries: the United States (*n* = 45; 65.20%), Netherlands (*n* = 12; 17.40%), Canada (*n* = 11; 15.90%), and Uganda (*n* = 1; 1.40%). We analyzed 66 quotes from 29 participants using an inductive thematic approach, and uncovered nine overarching themes: (1) Guidance and Mentorship, (2) Networking, (3) Training, (4) Time, (5) Funding, (6) Resources, (7) Support, (8) Sustainability, and (9) Institutional Limitations.

**Conclusion:**

Primary care research trainees face complex challenges such as limited time, funding, mentorship, and research skills, compounded by clinical demands and institutional barriers. Solutions include protected research time, structured mentorship, networking, and equitable institutional support. Future research should identify trainees’ priorities and develop actionable strategies to support primary care research trainees.

## Background

Global demand for primary care is accelerating, but investment in an inclusive, well-prepared primary care research workforce has not kept up. At the core of any research workforce are its trainees. Although trainees constitute a smaller proportion of the current research workforce, they represent its future in full. Yet, the specific needs and barriers of trainees pursuing primary care research are underexplored, as existing literature often focuses primarily on family medicine physicians and general practitioners (1–4). In the context of this study, we define a trainee as an individual currently engaged in formal education or postgraduate training, including students, residents, fellows, and other early-career learners (5). Recent initiatives have emphasized workforce inclusion, personnel diversity, trainee engagement, and accessible funding. These initiatives are critical to expanding the primary care research workforce and producing evidence that meaningfully improves the health and lives of patients and communities worldwide.

Persistent workforce shortages further compound the issue of limited production of quality primary care-orientated research (6–11). There is a dire need for primary care services in nearly every community internationally (12), creating a divide between time devoted to research and clinical care for primary care professionals (13). Despite clear potential to address gaps in protected time, funding and grant-writing support, mentorship, research staff, and access to tools and libraries, infrastructure to support primary care research professionals and trainees remains limited. These systemic challenges contribute to a self-fulfilling constraint that primary care research lacks adequate support and recognition (3), protected time (14), and funding (15–17). These concerns are not unfounded and have large impacts on both primary care trainees and established researchers, as well as the primary care field itself (18). Despite limited empirical literature on this topic, our perspectives as trainees and near-peers suggest that research-oriented trainees may view primary care as less conducive to research careers and instead pursue more traditionally research-intensive specialties (e.g., internal medicine subspecialties, surgery, neurology) (19, 20).

In addition to systemic challenges, it is important to understand additional reasons why trainees may not choose to pursue primary care research. To shift the narrative, we must examine why trainees are not pursuing primary care research, why the field fails to attract them, and how it can be transformed into a more compelling and viable option. This study aims to answer these questions through the assessment of training needs, available resources, and barriers encountered by existing trainees (clinical and non-clinical) in their pursuit of a career in primary care research. Through the identification of key areas for improvement, including skill development, access to research infrastructure and funding, and training, we aim to elucidate existing challenges and offer viable solutions.

## Materials and methods

### Study design

This cross-sectional study was conducted using an online survey between November 2024 and June 2025. The survey, administered via the REDCap platform, was in English and consisted of 33 items, including demographics. The survey featured both closed-choice and optional open-text questions, capturing quantitative and qualitative data. Survey items were developed inductively by the research team, based on lived experience as trainees and near-peers, and were informed by existing literature in non-trainee populations (21). We did not provide incentives or compensation for participation. The “*Assessing the Needs of Trainees*” section of the survey (Items 15-33) can be referenced in the Supplementary Appendix Table A. This exempt study was registered and approved by the Institutional Review Board (IRB) at the University of Missouri, Columbia, US (MU#2122129), with additional forms approved regarding international participation.

### Recruitment

The study sample included primary care trainees who were recruited using convenience sampling strategies. We recruited through professional primary care-based organizations and societies, including the NAPCRG (previously North American Primary Care Research Group), Association of Departments of Family Medicine (ADFM), Society of Teachers of Family Medicine (STFM), and Australian Association for Academic Primary Care (AAAPC). The survey was distributed via email and online community forums. At the 2024 NAPCRG annual conference, we recruited trainees to complete the online survey. After the conference, we emailed survey invitations using an internal NAPCRG list of trainees. To expand outreach, we asked faculty and principal investigators affiliated with NAPCRG to disseminate the survey to their students, programs, and institutions. Participants self-screened by identifying as trainees engaged in or planning to engage in primary care research. In this study, the definition of a trainee was purposely open-ended to capture a larger pool of trainee perspectives that encompasses both clinical (MD, MD/Ph.D, DNP, etc.) and research-focused (Ph.D, MA, etc.) trainees. Trainees self-reported their specific training under “Currently Enrolled Program” within demographics (see Table 1); however, no additional stratification or data collection occurred regarding the trainees’ year in program or training.

**TABLE 1:** Self-reported demographics.

Self-reported demographics	*N* [*n* % (n/69)]
Currently enrolled program	18 (26.1%) MD 17 (24.6%) medical residency 13 (18.8%) Ph.D. 5 (7.3%) MD/Ph.D. 4 (5.8%) master’s 1 (1.5%) bachelor’s 1 (1.5%) DNP 10 (14.5%) other 6 (8.7%) other: post-doc 2 (2.9%) other: fellow 1 (1.5%) other: GP trainee and Ph.D. candidate 1 (1.5%) other: medical resident and Ph.D. student
Current country of study	45 (65.2%) United States (US) 12 (17.4%) Netherlands 11 (15.9%) Canada 1 (1.4%) Uganda
International student status*	8 (11.6%)
Underrepresented in medicine (URiM)**	16 (23.2%)
First-generation college student	20 (29.0%)
Low-income or low-SES background	19 (27.5%)
Race	47 (68.1%) White or Caucasian 8 (11.6%) Asian 4 (5.8%) Black or African-American 5 (7.3%) other/not described 2 (2.9%) prefer not to disclose
Hispanic ethnicity	3 (4.4%) Hispanic or Latino 1 (1.4%) Prefer not to disclose
Gender identity	56 (81.2%) women 11 (15.9%) men 1 (1.4%) not described here 1 (1.4%) prefer not to disclose
Age range	38 (55.1%) 20–30 years of age 24 (34.8%) 31–40 years of age 5 (7.3%) 41–50 years of age 1 (1.4%) 51–60 years of age 1 (1.4%) decline to answer

*International student status is an international student who is enrolled in an educational institution outside their home country.

**Underrepresented in medicine (URiM) is defined by the American Association of Medical Colleges as “racial and ethnic populations that are underrepresented in the medical profession relative to their numbers in the general population.”

### Analysis

#### Quantitative analysis

Author AW performed quantitative analysis using RStudio for Windows (version 2025.05.1). The goal of this analysis was to explore factors that may influence trainees’ ability to do primary care research. As such, we have employed an exploratory data analysis approach to generate insights on patterns and relationships between the data. The outcomes of interest were trainees’ research skill confidence, training needs, access to resources, and challenges faced in primary care research. We included the following independent variables in our analysis: type of training program, country of study, gender, Under Represented in Medicine (URiM) as defined by the Association of American Medical Colleges (AAMC)’s 2024 definition (22), low-socioeconomic status (SES), international student status, and first-generation college student status. We used five-point Likert scale questions (from 0, not confident, to 4, very confident) to operationalize research skill confidence across seven domains. We calculated mean scores for each domain. Participants ranked the quality of mentorship on a five-point Likert scale (from 0, very poor, to 4, excellent). We operationalized all other variables of interest using discrete and multiple response formats. We calculated variable frequencies for initial descriptive analysis and analyzed response patterns for each question, using individual-level counts of selections.

We used Wilcoxon rank sum (Mann-Whitney U) to test mean confidence and quality scores, as well as counts of selections for multiple-select questions, against the independent variables. This non-parametric test was selected because Likert-scale data may not be normally distributed and Wilcoxon rank sum is robust to violations of normality assumptions. For bivariate analysis, we conducted Chi-square tests of each selection option in the multiple-select questions and the independent variables. When cell counts were sparse (frequencies < 5), a Fisher’s exact test was used to obtain exact *p*-values for more reliable inferences. Results were deemed statistically significant at the *p* < 0.05 level.

#### Inductive thematic analysis

We performed inductive thematic analysis manually using Microsoft Excel to identify overarching themes (23, 24). First, we categorized open-text responses by question. We analyzed the text verbatim, except for clarifying shorthand or to correct misspellings; these clarifications are indicated in brackets. Two coders (KTB, MB) analyzed the open-text responses independently using inductive codes derived from the literature and from our collective experience as trainees. Both coders are members of the NAPCRG Trainee Committee and are medical students in the United States (US). We calculated Cohen’s kappa to assess intercoder reliability (κ = 0.59; SD = 0.26); however, given the small sample of quotes, the statistic is highly sensitive to minor disagreements and may underestimate true agreement. Thus, we also calculated raw percent agreement to contextualize coder consistency. The two coders (KTB, MB) had an average of 90.22% raw percent agreement (SD = 6.05%), using the ReCal2 online tool (25). When consensus could not be reached, we applied the independent inductive codes from a third coder (KN).

## Findings

### Quantitative

Of the 99 responses received, 69 responses were included in the analysis after removing incomplete (*n* = 24) and ineligible (*n* = 6, self-identified as non-trainee) responses. The majority of participants were allopathic medical (MD) students, representing 28.1% of respondents (*n* = 18), followed by medical residents (*n* = 17; 24.6%). Participants spanned across four countries, including the US (*n* = 45; 65.2%), Netherlands (*n* = 12; 17.4%), Canada (*n* = 11; 15.9%), and Uganda (*n* = 1; 1.4%). We include demographic information on race, Hispanic ethnicity, gender identity, and age to showcase the overall diversity of survey respondents alongside the geographic and training diversity described above. The full list of self-reported demographic data is reported in Table 1.

### Mentorship

**FIGURE 1:**
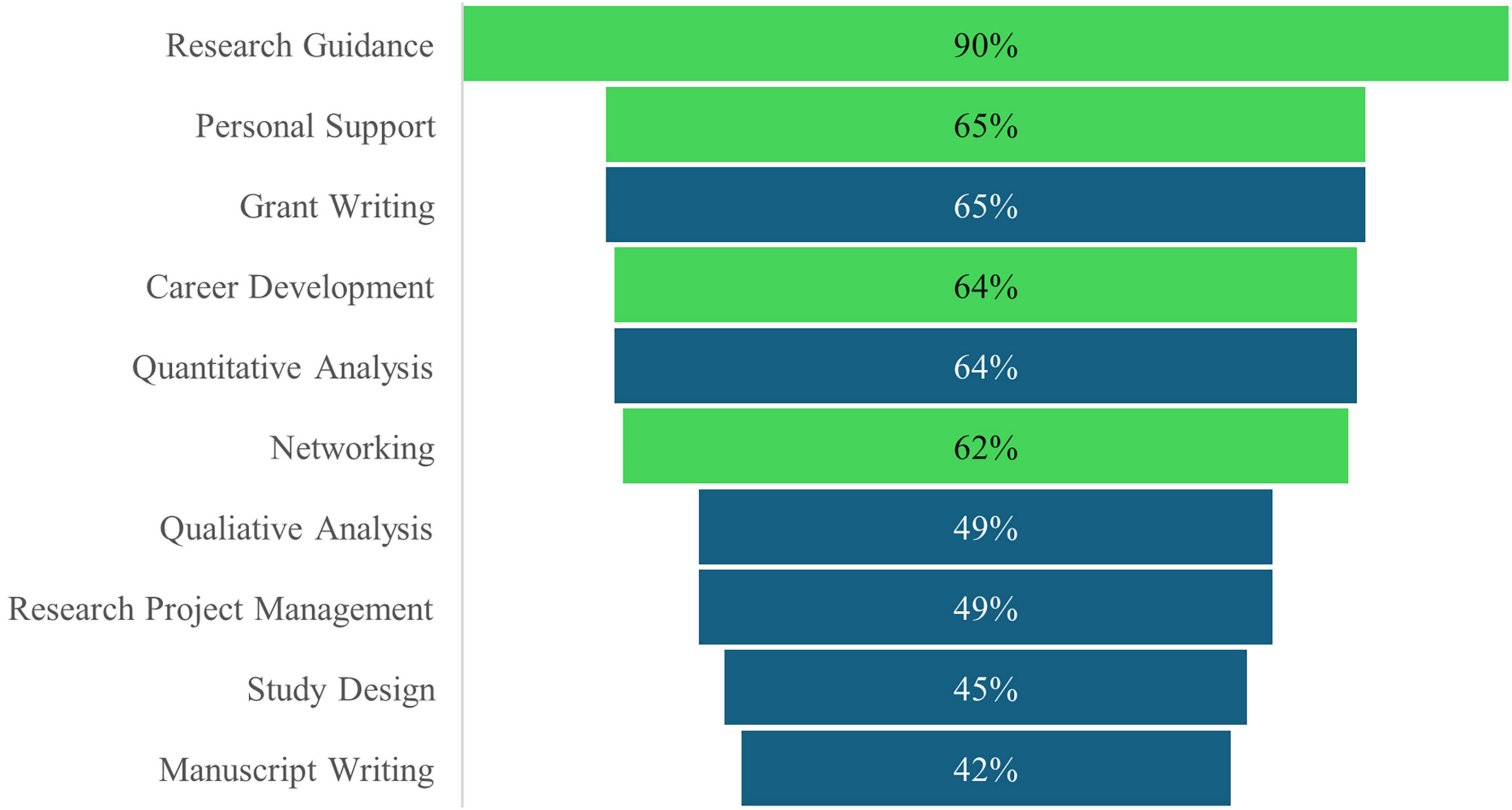
Reported mentorship and training needs of trainees (*n* = 69 responses). Mentorship needs and skills/training needs are denoted by green and blue, respectively.

All trainees reported at least one mentorship need (Figure 1 and Table 2). Fifty-seven participants (*n* = 57, 82.6%) said they have a research mentor who is formally involved in their training, such as an advisor, primary investigator, committee member, or supervisor. Additionally, one participant indicated that they did not have a formal research mentor; however, they went on to rate mentorship quality. Trainees generally rated the quality of their mentorship highly, with a mean score of 3.07 ± 0.14 on a five-point scale (0 = Very Poor, 4 = Excellent; *N* = 58). Participants often rated their mentorship as “Good” [*n* = 15, 25.9% (15/58)] or “Excellent” [*n* = 27, 46.5% (27/58)]. Only a small number of participants reported “Very Poor” [*n* = 2, 3.4% (2/58)] or “Poor” [*n* = 3, 5.2% (3/58)] mentorship quality. Approximately 16% (*n* = 11) of respondents marked this question as not applicable to them. There were no significant differences between the mean quality of mentorship scores when tested by Wilcoxon rank sum tests as reported by low-SES, URiM, First-Generation, or international students compared to their respective counterparts. Fisher’s exact test indicated a significant association (*p* = 0.044) between international student status and reporting career development as a helpful mentorship aspect. All international students (*n* = 8, 100%) reported this need, compared to 59.0% (*n* = 36/61) of non-international students. There was no association between helpful mentorship aspects and country of study or program.

**TABLE 2:** Full summary table of the self-identified needs, challenges, and available resources of trainees by domain and item selected (*n* = 69).

Domain	Item	*n*	% (n/69)
Mentorship needs	Research guidance	62	89.9%
	Career development	44	63.8%
	Networking	43	62.3%
	Personal support	45	65.2%
Challenges	Lack of time	51	73.9%
	Lack of mentorship	19	27.5%
	Quality of mentorship	10	14.5%
	Limited access to resources	28	40.6%
	Insufficient funding	32	46.4%
	Limited opportunities for collaboration	16	23.2%
	None of these	4	5.8%
Access to resources	Statistical software	40	58.0%
	Library access	61	88.4%
	Funding opportunities	27	39.1%
	Research assistants	22	31.9%
	Collaboration opportunities	36	52.2%
	Grant writing offices	11	15.9%
	Consistent lab/space	16	23.2%
	No resources	2	2.9%
Skills/training needs	Study design	31	44.9%
	Quantitative analysis	44	63.8%
	Qualitative analysis	34	49.3%
	Manuscript writing	29	42.0%
	Grant writing	45	65.2%
	Research project management	34	49.3%
	No additional training needed	6	8.7%

### Research skills

Trainees’ confidence in research-related skills varied across domains on a five-point scale (0 = Not Confident, 4 = Very Confident), presented fully in Table 3. Participants reported the highest levels of confidence in formulating a research question and presenting findings. High levels of confidence were also reported for conducting literature reviews, qualitative analysis, and writing for publication. Confidence was notably lower in grant writing, with nearly one-third (31.9%) of respondents reporting no confidence in this area. For the research skill of quantitative analysis, approximately 42% of participants indicated low confidence (ratings 0 or 1); the mean score was 2.93 (±0.15).

**TABLE 3:** Likert-scale confidence ratings in research skills among trainees to the question: “rate your confidence in the following research skills.”

Research skill	Mean score ± SE	Confidence rating (*n*, %)				
		0 = not confident	1	2	3	4 = very confident
Formulating a research question	3.75 ± 0.11	0, 0%	9, 13%	14, 20.3%	31, 44.9%	15, 21.7%
Conducting literature reviews	3.67 ± 0.12	1, 1.4%	10, 14.5%	14, 20.3%	30, 43.5%	14, 20.3%
Quantitative analysis	2.93 ± 0.15	8, 11.6%	21, 30.4%	16, 23.2%	16, 23.2%	8, 11.6%
Qualitative analysis	3.17 ± 0.15	7, 10.1%	16, 23.2%	14, 20.3%	22, 31.9%	10, 14.5%
Writing for publication	3.20 ± 0.15	7, 10.1%	15, 21.7%	15, 21.7%	21, 30.4%	11, 15.9%
Grant writing	2.14 ± 0.13	22, 31.9%	26, 37.7%	10, 14.5%	11, 15.9%	0, 0%
Presenting findings	3.75 ± 0.13	2, 2.9%	8, 11.6%	16, 23.2%	22, 31.9%	21, 30.4%

We used Wilcoxon rank sum test (Supplementary Appendix Table B) to compare the mean confidence ratings in research skills of low-SES, URiM, First-Generation students, and international students, and their counterparts. International students had significantly higher confidence in quantitative analysis (median = 4, IQR = 1), writing for publication (median = 4.50, IQR = 1.2), and grant writing (median = 3.0, IQR = 0.5) than non-international students (respectively: median = 3.0, IQR = 2; median = 3.0, IQR = 2.00; median = 2.0, IQR = 1.0). International students had a slightly higher confidence score in conducting literature reviews (median = 5.0, IQR = 1.25) compared to non-international students (median = 4.0, IQR = 1.00), but this difference was not significant (W = 146, *p* = 0.054). Finally, those with URiM status (median = 5.0, IQR = 1.25) had significantly higher confidence in presenting research findings than non-URiM trainees (median = 4.0, IQR = 1.00).

Needs for additional research skills training were also examined (Figure 1 and Supplementary Appendix Table B). In a series of Chi-square and Fisher’s exact tests, we found no statistically significant associations between any skill need and low-SES, URiM, international student status, or first-generation status. There were, however, significant associations between countries of study and self-reported needs for quantitative analysis (*p* < 0.001), qualitative analysis (*p* = 0.016), and manuscript writing skills (*p* = 0.002). Participants from the US and Canada were more likely to report needing quantitative analysis training (*n* = 34, 75.6%; *n* = 8, 72.7%, respectively) compared to those from the Netherlands (*n* = 1, 8.3%). Respondents from the US reported the highest need for qualitative analysis (*n* = 27, 60.0%) and manuscript writing (*n* = 25, 55.6%), followed by Canada (*n* = 4, 36.4% and *n* = 2, 18.2%) and Netherlands (*n* = 2, 16.7% and *n* = 1, 8.3%). The sole respondent from Uganda reported all three needs. There was also a significant association between program and need for more training in manuscript writing (*p* = 0.0005). This need for manuscript writing training was highest among MD students (*n* = 14, 78%), Bachelors (*n* = 1, 100%), DNP (*n* = 1, 100%), and post-graduate trainees (*n* = 1, 100%). In contrast, the need was much lower among PhD students (*n* = 2, 15%), those in more than one program (0%), and MD/Ph.D students (0%).

### Access to resources

When asked what resources they have access to, most trainees (*n* = 61, 88.4%) indicated they had library access, while only 11 (15.9%) participants indicated they had access to a grant writing office. Two participants indicated that they had access to no research resources. The most frequent response pattern was selecting only Library Access (*n* = 12, 17.4%). A full frequency table of the resources trainees indicated that they have access to can be found in Table 2.

A Wilcoxon rank sum test indicated a statistically significant difference in the number of resources selected between those who reported being of low-SES and those who did not (W = 681.5, *p* = 0.005). Respondents who were not of low-SES selected more resources (median = 3.00, IQR = 2.75) compared to those who reported low-SES (median = 2.00, IQR = 2.00). This indicates a significant disparity in access to resources, with trainees from low-SES backgrounds having access to fewer resources.

A series of chi-square and Fisher’s exact tests on resources available to trainees indicated statistically significant associations between country of study and access to both statistical software (*p* = 0.002) and research assistants (*p* = 0.001). Participants from Netherlands were most likely to report access to statistical software (*n* = 12, 100%) and research assistants (*n* = 8, 66.7%), whereas access to these resources was lowest among US respondents (*n* = 22, 48.9%; *n* = 8, 17.8%, respectively). Fisher’s exact test indicated a statistically significant association between program and access to funding opportunities (*p* = 0.026). Access to funding opportunities was most common among Ph.D. students (*n* = 7, 54%), students in “more than one program” (*n* = 3, 100%), and MD/Ph.D. students (*n* = 3, 60%). In contrast, access to funding was less common among MD students (*n* = 4, 22%), medical residents (*n* = 3, 18%), and not observed among Bachelors students (0%).

### Challenges faced in primary care

Regarding challenges faced by trainees in primary care, lack of time was cited most frequently, while quality of mentorship was least (Table 2). A series of Fisher’s exact tests indicated several statistically significant associations between participant demographics and challenges faced. Low-SES respondents were more likely to report “quality of mentorship” as a challenge (*n* = 6, 31.6%) compared to those without low-SES (*n* = 4, 8.0%; *p* = 0.022). International students were less likely to report “lack of time” (*n* = 3, 37.5%) than non-international students (*n* = 48, 78.7%; *p* = 0.024) but more likely to report “insufficient funding” (*n* = 7, 87.5% vs. *n* = 25, 41.0%; *p* = 0.021). Program was statistically significantly associated with the likelihood of reporting several primary care challenges: specifically, lack of time (*p* = 0.0009), lack of mentorship (*p* = 0.049), and access to resources (*p* = 0.049). Lack of time was reported most frequently by medical residents (*n* = 17, 100%), MD students (*n* = 15, 83%), and students in more than one program (*n* = 3, 100%). Lower proportions were observed among Masters students (*n* = 1, 25%), Bachelors (0%), and post-graduate trainees (0%). Lack of mentorship was most commonly reported by Bachelors (*n* = 1, 100%), DNP (*n* = 1, 100%), MD students (*n* = 8, 44%), and Masters students (*n* = 2, 50%). The challenge was less common among Ph.D. students (*n* = 1, 8%), and absent in all other trainees. Access to resources was reported most frequently by MD students (*n* = 13, 72%) and Ph.D. students (*n* = 5, 38%). Lower proportions were seen among students in more than one program (*n* = 1, 33%) and absent in all other trainees. Finally, there was a significant association between gender and reporting “lack of mentorship” (*p* = 0.046). Women were more likely to report this challenge (*n* = 19, 33.9%) compared to men, other, or not disclosed (all 0%). There was no association between challenges faced by trainees and country of study.

### Qualitative

We had a total of 66 optional open-text responses from 29 participants [42.0% of total included sample size (29/69)]. We identified a total of nine overarching themes from an inductive thematic approach, as described in the Methods: (1) Guidance and Mentorship, (2) Networking, (3) Training, (4) Time, (5) Funding, (6) Resources, (7) Support, (8) Sustainability, and (9) Institutional Limitations. The most common theme response was Guidance and Mentorship, appearing across all four questions. All themes categorized by question posed can be found in Table 4.

**TABLE 4:** Qualitative responses organized by question and corresponding themes, presented in order from most to least represented.

What additional support do you need from your mentor or research team? What aspects of mentorship have been the most helpful to you?	*n*/18 (%)
Guidance and Mentorship (i.e., planning for the future, next steps of career, research guidance)	13 (72.2%)
Time (i.e., limited availability, dedicated research time, and the push-pull between clinical and research responsibilities)	4 (22.2%)
Support (i.e., needs support from institution, project management)	4 (22.2%)
Networking (i.e., building supportive systems beyond the institution, fostering connections, and collaborating with peers)	2 (11.1%)
Training (i.e., more training needed, research methodology training)	2 (11.1%)
Funding (i.e., grant writing, low-salary, limited grants)	1 (5.6%)
Institutional Limitations (i.e., limited primary care prioritization by institution, lack of investment by field)	1 (5.6%)
**What resources or opportunities do you feel are lacking in your current research environment?**	***n*/16 (%)**
Funding (i.e., grant writing, low-salary, limited grants)	7 (43.8%)
Resources (i.e., no physical space, limited equipment, no libraries)	4 (25.0%)
Time (i.e., limited availability, dedicated research time, and the push-pull between clinical and research responsibilities)	4 (25.0%)
Training (i.e., more training needed, research methodology training)	3 (18.8%)
Support (i.e., needs support from institution, project management)	3 (18.8%)
Institutional Limitations (i.e., limited primary care prioritization by institution, lack of investment by field)	3 (18.8%)
Guidance and Mentorship (i.e., planning for the future, next steps of career, research guidance)	2 (12.5%)
Networking (i.e., building supportive systems beyond the institution, fostering connections, and collaborating with peers)	2 (12.5%)
**What support would help you overcome these challenges?**	***n*/21 (%)**
Funding (i.e., grant writing, low-salary, limited grants)	8 (38.1%)
Guidance and Mentorship (i.e., planning for the future, next steps of career, research guidance)	6 (28.6%)
Time (i.e., limited availability, dedicated research time, and the push-pull between clinical and research responsibilities)	5 (23.8%)
Institutional Limitations (i.e., limited primary care prioritization by institution, lack of investment by field)	5 (23.8%)
Networking (i.e., building supportive systems beyond the institution, fostering connections, and collaborating with peers)	4 (19.1%)
Support (i.e., needs support from institution, project management)	3 (14.3%)
Training (i.e., more training needed, research methodology training)	2 (9.5%)
Sustainability (i.e., burnout, wellness, stress)	1 (4.8%)
**Is there anything else you would like to share about your needs as a primary care research trainee?**	***n*/11 (%)**
Funding (i.e., grant writing, low-salary, limited grants)	6 (54.6%)
Support (i.e., needs support from institution, project management)	4 (36.4%)
Guidance and Mentorship (i.e., planning for the future, next steps of career, research guidance)	3 (27.3%)
Time (i.e., limited availability, dedicated research time, and the push-pull between clinical and research responsibilities)	3 (27.3%)
Sustainability (i.e., burnout, wellness, stress)	3 (27.3%)
Institutional Limitations (i.e., limited primary care prioritization by institution, lack of investment by field)	3 (27.3%)
Resources (i.e., no physical space, limited equipment, no libraries)	2 (18.2%)
Networking (i.e., building supportive systems beyond the institution, fostering connections, and collaborating with peers)	1 (9.1%)

Some codes overlap and are co-coded across themes, and certain themes are not represented within specific questions. Text items in bold indicate the open-ended and free-text question posed to participants in the online survey.

#### Theme 1: Guidance and Mentorship

*Guidance and Mentorship* emerged as a main theme in every open-text question posted to participants. Additionally, we identified several subthemes, based on implications from participant responses such as: planning for the future, next steps of career, and research guidance. A sample of quotes that exemplify the theme of *Guidance and Mentorship* are provided below:

*“Research is especially important for pursuing competitive specialties in medicine. So having a mentor who understands this and is determined to help get published work, not just the experience.” [ID#89, Medical (MD) Student in the US]*

*“Career development and research guidance since the world is heading toward research and project management.” (ID#91, Bachelor’s Student in Uganda)*

*“At first, research and personal guidance was most important to me. As the years went by, career development played a bigger role.” (ID#85, Medical Resident and Ph.D. Student in Netherlands)*

#### Theme 2: Networking

Respondents frequently emphasized the importance of building supportive systems beyond their institution, fostering connections, and collaborating with peers, which were coded under the theme of Networking. Networking was often coded in addition to the theme Guidance and Mentorship.

*“Real networking-not simply a cocktail hour. Hearing how reviewers feel about grants they receive, not simply being told what elements to create. Pilot funding that would allow me to become competitive.” (ID#31, Post-doctoral Student in the US, in regards to overcoming challenges)*

*“Proper primary care connections through my medical school.” [ID#75, Medical (MD) Student in the US]*

#### Theme 3: Funding

Another prominent theme that was described by participants was Funding. This theme relates primarily to limited grant writing knowledge, low-salaries, access to funding, and limited grant opportunities. Four respondents expressed feeling that Funding was lacking or limited in their current research environment. Another respondent highlighted the need for grant writing and clear application pathways to find funding opportunities. Multiple respondents cited difficulties obtaining financial support for primary care, and its personal impact.

*“Everyone tells me that if I become a researcher I will need to fund 100% of my time. How am I supposed to do that? And if I do, and all my time is committed to projects, how do I have time to write or seek the next funding opportunity?” (ID#31, Post-doctoral Student in the US)*

*“I have done my research with my own resources, and although I have been very successful, I haven’t received any funding. Being an external Ph.D. student living in another country has made it difficult to apply for grants, difficult to connect well with all supervisors, and unavailable for me. This should be informed to students when they apply for a position.” (ID#30, MD/Ph.D. in Netherlands)*

*“We also need to have access to funds that enable us to pay ourselves a reasonable living wage and have enough left over to conduct our research. It’s very hard to do research outside of your supervisor’s grant. Having a supervisor with access to money and funding is great, but I also think trainees should have more independence to manage their research portfolio, otherwise we’re not going to have experience when we move into faculty or teaching positions.” (ID#95, Ph.D. Student in Canada)*

*“The department needs more funding, so that folks that have the knowledge base can mentor trainees like me.” [ID#25, Doctor of Nursing Practice (DNP) Student in the US]*

#### Theme 4: Time

The theme Time describes the need for protected or dedicated time. Time is also described as the need for time away from clinical or other obligations to focus on primary care research. Three participants specifically highlighted the push-and-pull between clinical responsibilities and research.

*“A [big] issue in primary care is the time. Even with great mentors, library access, some research support, there’s just no time. You see 12–20+ patients in a day, addressing multiple issues per visit, the documentation takes time after the visit, the lab reviews, forms to be filled out, [prescriptions] to refill, and the patient portal messages never seem to end…” (ID#E, Medical Fellow in the US)*

*“Time away from clinical duties structured for research work.” (ID#19, Medical Resident in the US)*

*“Protected time for research in residency.” [ID#5, Medical (MD) Student in the US]*

*“Difficult to find time as a part-time student juggling a family.” (ID#99, Ph.D. Student in Canada)*

#### Theme 5: Sustainability

Time was frequently coded in addition to the theme of Sustainability (i.e., burnout, wellness, stress). The following quotes best highlight this:

*“The exhaustion at the end of a 10-h clinic day doesn’t lend itself well to research. If you have a non-clinical day, you’re likely catching up.” (ID#E, Medical Fellow in the US)*

*“Researchers who aren’t also clinicians just don’t get that, without time and wellness in programs, research for resident doctors just is busy work. How can it compare to the needs of your patient who is dying or the patient who needs you to spend an hour on the phone to get their insulin approved. When you’re constantly overworked and exhausted, trying to make time for mandatory research projects just fails. And when we do make it happen, we try to do the bare minimum because we’re constantly in survival mode in a background of constantly traumatizing events. If you want us to do quality research projects, we need to have it as dedicated elective time (like at least two weeks straight at a time) during business hours with structured, real time support and access to mentors like it would be in graduate school. Or having a separate research year like in some residencies.” (ID#19, Medical Resident in the US)*

*“Literally time and not being so overworked and burned out by clinical training.” (ID#19, Medical Resident in the US)*

#### Theme 6: Institutional Limitations

Institutional Limitations was often described as limited primary care prioritization by institution and lack of investment in trainees by the field of primary care. The institutional deprioritisation negatively impacts primary care research trainees and undermines opportunities for them to develop into independent researchers.

*“Increased awareness of issues pertinent to the primary care population and interest from administrators and institutions.” [ID#35, Medical (MD) Student in the US]*

*“The school prioritizes research for students in non-primary care specialties*… *It has also been a pattern that career development connects those interested in surgical specialties with research but offers minimal to no guidance toward those interested in primary care.” [ID#43, Medical (MD) Student in the US]*

*“At my program, there is a research project that we complete, but not until our second or third year…there aren’t a lot of opportunities to get involved in research early.” (ID#23, Medical Resident in the US)*

*“I am able to research because of scholar months in Washington, DC and AAFP. My community residency program could not support my desires for research without these opportunities.” [ID#5, Medical (MD) Student in the US]*

#### Theme 7: Training

Training reflects primary care trainees’ need for more formal instruction and curriculum, particularly in research methodology. This theme frequently co-occurred with codes related to Institutional Limitations. Respondents discussed the need for curricular improvements and critiqued institutional priorities—or lack thereof—regarding primary care.

*“I don’t think this is specific to my mentor, but rather my medical school as a whole, because I feel like I’ve received very little formal training in biostatistics.” [ID#64, Medical (MD) Student in the US]*

*“More structured curricula and didactics around research. More dedicated time.” (ID#48, Medical Resident in the US)*

*“More quantitative analysis training for learners (specifically clinicians who don’t receive as much training).” [ID#5, Medical (MD) Student in the US]*

#### Theme 8: Resources

Resources was a theme describing what participants underscored as lacking in their current academic programs. These resources ranged from physical spaces, libraries, access to ancillary courses, and software. Moreover, one respondent stated that they were unsure of the resources available and limited opportunities to get involved in primary care research.

*“.I’m a little unsure of all of the resources, and there aren’t a lot of opportunities to get involved in research early.” (ID#23, Medical Resident in the US)*

*“Formal ancillary courses related to research. All training is mostly in real time.” [ID#89, Medical (MD) Student in the US]*

*“Grant writing, statistical software, and financial opportunity*… *Equipment, since now [I] am [accessing] these using a hand phone, and financial support.” (ID#91, Bachelor’s Student in Uganda)*

#### Theme 9: Support

Lastly, respondents discussed the need for support from their institutions and in project management. The theme of Support frequently overlapped (via additional applied codes) with Funding and Guidance and Mentorship, exemplifying the multifaceted nature of support needs among primary care research trainees.

*“I need additional support for data analysis.” [ID#5, Medical (MD) Student in the US]*

*“Only two of my four supervisors have been really helpful in the whole learning process of my PhD. What I am missing is support for planning my future although I have conducted very successful research - and research funding.” (ID#30, MD/Ph.D. Student in Netherlands)*

*“Funds/structures to support transitioning from trainee to independent researcher.” (ID#47, Post-doctoral Fellow in Canada)*

*“[My mentors] are always available for questions, never feeling like I bother them. They respect my ideas. Perhaps, I would need a bit more personal support.” (ID#87, MD/Ph.D. Student in Netherlands)*

### Integration of results

**FIGURE 2:**
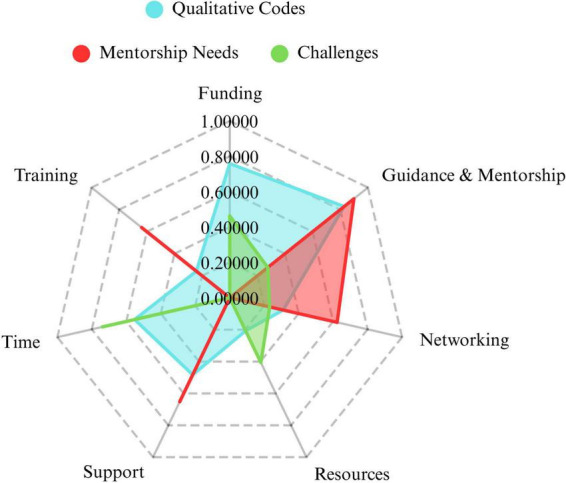
Radar chart of the frequency proportions of qualitative themes (codes) and quantitative (mentorship needs and challenges) to highlight areas of overlap and divergence, as a conceptual illustration.

To compare and integrate the results, we have generated a radar chart as a conceptual visualization of the overlap of results within both qualitative and quantitative data (Figure 2). Based on the coders’ descriptions of the themes, quantitative items were matched where possible with qualitative themes. Not all questions that were asked in the quantitative part of the survey had a parallel concept that was inductively found within the qualitative data, therefore the joint display is limited to data on mentorship needs, challenges faced by trainees, and seven of the nine themes (Funding, Training, Time, Support, Resources, Networking, Guidance and Mentorship). To create the illustration, we calculated the quantized frequencies of each question item from the quantitative data and the frequencies of each distinct code within the qualitative data. Proportions were calculated for each theme or item included. This conceptual chart demonstrates that our quantitative and qualitative results converged the most on the concepts of guidance and mentorship, and networking.

## Discussion

Primary care research trainees often face complex needs, yet little empirical research has explored these issues. These complexities span several domains, including research skills, access to resources, and intersections of program type (PhD, MD, DNP, etc.). Of the existing literature, one study conducted by Ameh et al. (25) reported similar findings, including analysis of time, access to resources, and research skill confidence. In their study on early-career family physicians in sub-Saharan Africa, they found that time constraints due to work overload, conflicting responsibilities, and/or lack of protected time, was a barrier to research (26). A multitude of gaps exist in research training in primary care, including lack of: protected time, funding, training, research skills, and supervision or support (21). Highlighting the need for protected time as critical aligning with our finding that time was a frequently cited challenge among participants.

Participants articulated the push-and-pull dynamic between clinical responsibilities and research, as supported in the literature (26). Intersecting with time, qualitative responses embody the idea of Sustainability in relation to burnout and stress (27), which has been correlated with a loss in productivity (28, 29). One participant says this best: “When you’re constantly overworked and exhausted, trying to make time for mandatory research projects just fails. And when we do make it happen, we try to do the bare minimum because we’re constantly in survival mode in a background of constantly traumatizing events…” (ID#19, Medical Resident in the US). As a result, clinically based trainees often experience burnout early—before fully establishing their careers, let alone engaging as active researchers (30, 31).

Trainees consistently identified Funding as a significant barrier. Nearly one-third lacked confidence in grant writing, and only 15.9% endorsed access to a grant writing office. Fewer than half had access to any research funding, with international students disproportionately reporting insufficient funding. Qualitative responses echoed these barriers, with one-third of all quotes referencing financial struggles, limited grant pathways, and inadequate institutional support. A recent study on Canadian primary care researchers suggests that early-career researchers in primary care (and as such, trainees) are less successful when compared to established researchers (32). Low grant writing confidence reflects not only a skills gap but also limited accessible infrastructure, leaving trainees–especially international or low-SES students–at a systemic disadvantage when competing for scarce resources. Primary care research trainees are left to navigate the complexities of grant writing with little support, a critical skill essential to becoming a successful independent researcher (33). Funding challenges in primary care (32) research are compounded by the lack of dedicated grant writing offices and limited training in grant writing (21).

Approximately 42% of respondents reported low confidence in quantitative analysis, pronounced among US and Canadian trainees and those in non-research oriented programs. Confidence levels were generally stronger in Ph.D. and MD/Ph.D. programs, suggesting that research-intensive curricula provide better preparation. These findings indicate that trainees in graduate or dual-degree programs may develop stronger research skills than those in clinical programs alone. As expected, trainees in research-oriented programs will develop greater research proficiency; these findings highlight the need for clinical programs to improve skill-based research curriculum. Qualitative responses reinforced this pattern, with participants frequently citing limited biostatistics instruction and quantitative training. Ameh et al. (25) reported similar findings, identifying limited training in research analysis as a common barrier among trainees and early-career family physicians. However, their study highlighted the need for training in qualitative (26), while our trainee population demonstrates a greater need for training in quantitative methods.

A prevalent theme throughout quantitative and qualitative trainee responses was Mentorship and Guidance. Trainees indicated that while good mentorship exists in primary care research, there is an unmet need for research guidance and career development. Enhancing mentorship that fosters both scientific independence and career growth is essential (34–36) for building and sustaining a strong primary care research workforce. At the same time, opportunities for mentors to enhance support and connect with trainees grow. Trainees described evolving mentorship needs, accentuating the complex nature of guidance over time, with one respondent emphasizing the value of research guidance and career development.

### Solutions

Trainees identified challenges and unmet needs, they pointed to meaningful opportunities for solutions, sharing thoughtful qualitative insights that underpin barriers, while offering practical solutions. One respondent states *“lack of participation from the field” (ID#83, MD/Ph.D. Student in Netherlands)* as a challenge. Importantly, solutions call for active support from faculty mentors, academic institutions, and professional societies.

Institutional change is critical. As discussed above, there is a push-and-pull dynamic between high-demand clinical responsibilities and research opportunities for clinical trainees. Hospital systems and academic centers prioritize patient care above all else, incentivized by financial considerations, which limits investment in trainees. As a result, primary care clinician-scientist trainees risk burnout. Furthermore, the focus remains physician-centric in primary care, leaving research trainees underserved. To move forward, the field must adopt solutions that support all primary care research trainees equitably, ensuring programs are both impactful and inclusive.

One proposed solution is to offer clinical-based trainees dedicated time for research (37, 38). In the US, efforts have begun for physician-scientists. For example, the ADFM proposed a Family Medicine–Physician Scientist Pathway (FM-PSP) (39, 40) in 2017, with a pilot launched in 2019 with seven participating residency programs (40). Although an important first step, urgent expansion of primary care research-focused pathways is needed. Several formal Physician-Scientist Training Programs (PSTPs) exist for internal medicine residency programs, but many internal medicine residents subspecialize outside of primary care (41, 42). PSTPs, beyond the FM-PSP pilot, appear to be absent in family medicine. This absence may signal to medical students, even before matching, that primary care is less invested in cultivating physician-scientists than other specialties. In Canada, opportunities for primary healthcare trainees to receive further training exist within programs such as Transdisciplinary Understanding and Training on Research–Primary Health Care (TUTOR-PHC), which takes an interdisciplinary approach training primary health care researchers (43, 44). TUTOR-PHC also offers early-career researchers, clinicians, and decision-makers opportunities for skill building via collaboration (45). Establishing a narrative that primary care is a research-focused specialty is essential to attract the next generation of clinician-scientists and researchers; this requires concrete action, such as expanding access to and adopting initiatives modeled after the FM-PSP and TUTOR-PHC.

Another area for organizational improvement is the facilitation of connections between mentors and trainees. Due to the breadth of topics that fall under primary care research, it is essential to match trainees with mentors who share their research interests, while providing the training and guidance that trainees identify as necessary (43). Among the solutions proposed by participants, one (ID #43) advocated for an up-to-date list of primary care researchers willing to mentor students, while another (ID #11) suggested creating a formal mentorship program, through a professional society like NAPCRG. Collectively, these suggestions underscore the importance of structured, high-quality networking and highlight mentors’ willingness to foster such connections (45). A key recommendation is the development of a centralized repository of faculty available to mentor trainees, both nationally and internationally. Participants advocate for a platform that facilitates matches between trainees and mentors, based on shared research topics, skills, mentorship needs, and available opportunities. This represents a significant opportunity for professional societies to address an urgent trainee need by developing and maintaining an open-access mentorship platform.

Further guidance for mentors includes serving as strong advocates for the needs of trainees—such as protected research time, funding, and training in research skills. While resources for mentor development exist, these may need to be modified to best support primary care research trainees. Mentorship that extends beyond career development and project guidance needs to encompass personal support as trainees navigate the duality of advancing their careers and managing life. Future research should aim to identify trainees’ training priorities and design actionable solutions, including the allocation of appropriate resources, to support their development.

### Limitations

This study has several limitations that should be considered. Our use of a convenience sample and a recruitment strategy that leveraged the personal and professional network of authors, poses a risk of institutional bias in the responses we received. Furthermore, the sample size of the recruited participants is small (*n* = 69) with representation from a few countries, limited response rate from nursing students and no responses from osteopathic medical trainees. More self-identified women (*n* = 56, 81.2%) compared to other genders [men (*n* = 11, 15.9%), “other” (*n* = 1, 1.4%), and prefer not to disclose (*n* = 1, 1.4%)], responded to the survey which may skew any gender-based analysis we performed. As a result, the generalizability of this study is limited.

Of our survey respondents, five respondents self-identified as White and URiM. We are unsure if these five participants had ethnic identities not captured in the survey options or if they identified with other aspects of diversity (i.e., low-SES, first-generation college students, etc.) beyond the AAMC definition, which represents an U.S. American-centric interpretation. For the purpose of analysis and honoring these self-reported identities, we decided to keep these responses.

While the sample size for our study was modest, some of our subgroup analyses were done with very small and uneven sample sizes resulting in low statistical power. Because this study was exploratory in nature, we had a high number of tests, which may also create a multiple comparisons problem. This means there is greater risk of encountering false positives, or type I error. We decided to forego a *p*-value adjustment on these tests due in part because of the low statistical power, but also because of the high number of tests which would create higher risk for false negative results. Ultimately, statistical results should not be considered confirmatory and are only intended to provide preliminary insight into the topic area.

Additionally, the quotes were captured in a survey and optional. As such, we were not able to clarify or further investigate participants’ attitudes or opinions toward each question offered. This limits our ability to represent all data in the joint display because the concepts from quantitative and the qualitative do not align perfectly. However, this is expected in an exploratory design. In follow-up studies, more rigorous qualitative design methods should be employed including focus group or interview sessions, ensuring there are parallel measures between quantitative and qualitative concepts so that the data may show better convergence or divergence.

Of note, participants were already decided on or interested in pursuing primary care research and, as such, this study lacks the perspective of trainees who have opted for other specialties and research fields. Additional studies regarding trainees who elect to pursue non-primary care research fields and why they make this choice could elucidate how to promote primary care as a research specialty to trainees.

### Strengths

Our study features several strengths, particularly its use of a mixed-methods approach to data analysis. Using both quantitative and qualitative measures allowed us to cross-validate the findings and increased credibility. Further, triangulation of the qualitative data allowed formation of a nuanced view of the quantitative data. Our survey was co-designed by current trainees and near-peers, leveraging their lived experiences to inform the question design. Data analysis was conducted by current trainees and near-peers bringing nuanced perspectives that enriched our findings.

The online survey format allowed for wide distribution resulting in participation from trainees across four countries, and subsequently four distinct health systems and training nuances. Our findings may indicate that the overarching and broad problems trainees perceive in primary care research are prevalent across certain countries. Additionally, our study had appropriate diversity of participants across numerous domains, as described by self-identified URiM, low-SES, international student, and first-generation to attend college.

## Conclusion

Primary care research trainees across the world face a similar range of multifaceted challenges that can shape their future careers. Limited time, funding, mentorship, grant-writing resources, and research-methodology skills—compounded by increasing clinical demands and institutional barriers—hinder the development of trainees into successful primary care researchers. Addressing these issues requires protected and dedicated research time, structured mentorship, networking opportunities, and equitable institutional support for primary care research. Future studies should explore trainees’ priorities and develop actionable, well-resourced strategies to strengthen the primary care research workforce as global demand for primary care continues to grow.

## Data Availability

The datasets presented in this article are not readily available because to protect identities of the people who participated, only de-identified and redacted information (i.e., names, locations, organization, etc. will NOT be provided) and the dataset will be only be accessible upon request and approval. Requests to access the datasets should be directed to ktbrgt@health.missouri.edu.
