# Supplementary material for: Turning problems into progress for primary care research trainees: a mixed-methods analysis of an online cross-sectional survey
Source: Front Med (Lausanne). 2026 May 15;13:1786438. doi: 10.3389/fmed.2026.1786438 (PMC13218949; doi:10.3389/fmed.2026.1786438)
Supplement: Supplementary file 1 [file Supplementary_file_1.docx]

***Table A****: Assessing the Needs of Trainees section of the survey (Items 15-33).*

| **Item #** | **Question** | **Additional Notes** |
| --- | --- | --- |
| 15 | How many years of experience do you have in primary care research?   - None - 1-2 years - 3-5 years - More than 5 years |  |
| 16 | Rate your confidence in the following research skills:  Formulating a research question | Likert Scale: 0 (Not Confident) - 4 (Very Confident) |
| 17 | Rate your confidence in the following research skills:  Conducting literature reviews | Likert Scale: 0 (Not Confident) - 4 (Very Confident) |
| 18 | Rate your confidence in the following research skills:  Data analysis (quantitative) | Likert Scale: 0 (Not Confident) - 4 (Very Confident) |
| 19 | Rate your confidence in the following research skills:  Data analysis (qualitative) | Likert Scale: 0 (Not Confident) - 4 (Very Confident) |
| 20 | Rate your confidence in the following research skills:  Writing manuscripts for publication | Likert Scale: 0 (Not Confident) - 4 (Very Confident) |
| 21 | Rate your confidence in the following research skills:  Grant writing | Likert Scale: 0 (Not Confident) - 4 (Very Confident) |
| 22 | Rate your confidence in the following research  skills:  Presenting research findings | Likert Scale: 0 (Not Confident) - 4 (Very Confident) |
| 23 | What specific research skills would you like more training in? (Check all that apply)   - Study design - Data analysis (quantitative) - Data analysis (qualitative) - Manuscript writing - Grant writing - Research project management - No additional training needed |  |
| 24 | Do you currently have a research mentor? (A research mentor is someone formally involved in your training, such as an advisor, primary investigator, committee member, supervisor, etc.)   - Yes - No |  |
| 25 | If yes, how would you rate the quality of mentorship  you receive? | Likert Scale: 0 (Very poor) - 4 (Excellent) |
| 26 | Are you currently receiving funding from your  department or supervisor to support your education or research activities?   - Yes - No - N/A |  |
| 27 | What aspects of mentorship are most helpful to you? (Check all that apply)   - Research guidance - Career development - Networking opportunities - Personal support |  |
| 28 | What additional support do you need from your mentor or research team? What aspects of mentorship have been the most helpful to you? | Open text response, *optional* |
| 29 | What types of resources do you have access to for  conducting research? (Check all that apply)   - Statistical software (e.g., SPSS, R, Stata) - Library access - Funding opportunities - Research assistants - Collaboration opportunities - Grant Writing Offices - Consistent Lab or Clinical Space - None of these |  |
| 30 | What resources or opportunities do you feel are  lacking in your current research environment? | Open text response, *optional* |
| 31 | What challenges do you face in pursuing primary care research? (Check all that apply)   - Lack of time - Lack of mentorship - Quality of mentors/mentorship - Limited access to resources - Insufficient funding - Limited opportunities for collaboration - None of these |  |
| 32 | What support would help you overcome these challenges? | Open text response, *optional* |
| 33 | Is there anything else you would like to share about  your needs as a primary care research trainee? | Open text response, *optional* |
