# Supplementary material for: Turning problems into progress for primary care research trainees: a mixed-methods analysis of an online cross-sectional survey
Source: Front Med (Lausanne). 2026 May 15;13:1786438. doi: 10.3389/fmed.2026.1786438 (PMC13218949; doi:10.3389/fmed.2026.1786438)
Supplement: Supplementary file 2 [file Supplementary_file_2.docx]

***Table B****: Statistically significant group comparisons of trainee research skill confidence*

| **Research Skill** | **Group Comparison** | **Test Result** |
| --- | --- | --- |
| Quantitative analysis | International > Non-Int. | W = 94.5, p = 0.004 |
| Writing for publication | International > Non-Int. | W = 127, p = 0.025 |
| Grant writing | International > Non-Int. | W = 111, p = 0.009 |
| Presenting findings | URiM > Non-URiM | W = 278.5, p = 0.032 |
